# Combined aggregation induced emission (AIE), photochromism and photoresponsive wettability in simple dichloro-substituted triphenylethylene derivatives†
†Electronic supplementary information (ESI) available: Synthetic procedures, experimental details and supplemental figures. See DOI: 10.1039/c6sc01205a


**DOI:** 10.1039/c6sc01205a

**Published:** 2016-04-26

**Authors:** Depei Ou, Tao Yu, Zhiyong Yang, Tiangang Luan, Zhu Mao, Yi Zhang, Siwei Liu, Jiarui Xu, Zhenguo Chi, Martin R. Bryce

**Affiliations:** a PCFM Lab , GDHPPC Lab , Guangdong Engineering Technology Research Center for High-performance Organic and Polymer Photoelectric Functional Films , State Key Laboratory of OEMT , School of Chemistry and Chemical Engineering , Sun Yat-sen University , Guangzhou 510275 , China . Email: ceszy@mail.sysu.edu.cn ; Email: chizhg@mail.sysu.edu.cn; b MOE Key Laboratory of Aquatic Product Safety , School of Life Sciences , South China Sea Bio-Resource Exploitation and Utilization Collaborative Innovation Center , Sun Yat-sen University , Guangzhou 510275 , China . Email: cesltg@mail.sysu.edu.cn; c Department of Chemistry , Durham University , Durham DH1 3LE , UK

## Abstract

A dichloro-substituted triphenylethylene derivative (TrPECl_2_) with aggregation-induced emission (AIE), photochromism and photoresponsive wettability has been synthesized. The new compound shows fast-response photochromic behaviour with good ON/OFF repeatability by utilizing a proposed stilbene-type intramolecular photocyclization in the solid state. Compared with the more usual diphenylethylene derivatives, the photochromic properties of the triphenylethylene derivative are much more striking and easier to achieve. The triphenylethylene derivative also displays AIE properties leading to strong fluorescence in the solid state. Therefore, both the ultraviolet-visible absorption and fluorescence emission are drastically changed during the photochromic processes. Furthermore, the morphology of the TrPECl_2_ microcrystalline surface could be controlled by irradiation. The wettability of the surface could be drastically decreased with contact angles of a water droplet changing from 73° to 118°. The triphenylethylene derivative with a simple molecular structure is, therefore, attractive for multifunctional materials.

## Abstract 2

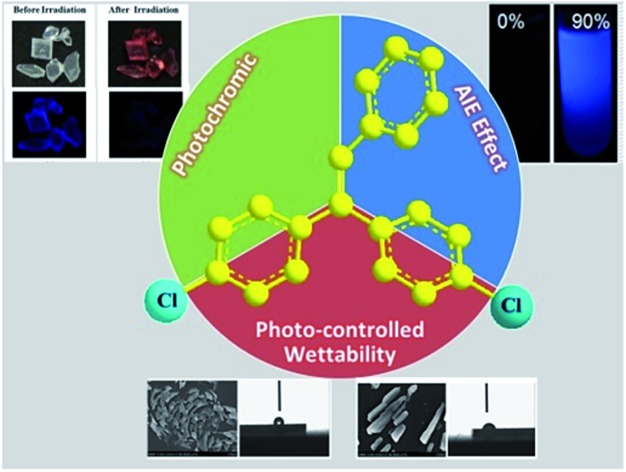
We present AIE, photochromism and photoresponsive wettability properties in a simple dichloro-substituted triphenylethylene molecule.

## Introduction

Photochromic materials are a type of photoresponsive compounds which undergo reversible chemical transformations between two (or more) well-defined states which have different optical properties. In recent years they have attracted much attention for their fundamental properties and for their emerging applications in security markings, optical shutters, photo-switchable molecular devices and optical memory storage systems.^1^,^2^ Accordingly, a wide range of photochromic systems have been developed, including stilbene- and azo-containing compounds,^3^ spirooxazine,^4^ spiropyran^5^ and diarylethene^6^ (especially dithienylethene), and dihydroazulene derivatives.^7^

Photochromic materials are also investigated as topographical change materials.^8^ Furthermore, the wettability of the surfaces could be changed with morphology control by irradiation.^8^,^9^ In previous studies, topographical change properties of diarylethene derivatives have been systematically investigated by Uchida,^10^ Kobatake,^11^ and other groups.^12^ Spiropyran and azo-containing compounds have also been used as topographical change materials.^13^ Widespread applications are still restricted by the unstable surface properties, the demands of long irradiation time and complex synthetic procedures. Therefore, fast-response photochromic systems with simple molecular structures obtained from readily-available starting materials are in high demand.

In this regard, diphenylethylene derivatives have been investigated. However, their photochromic properties are difficult to observe as the key cyclization reaction occurs only for the *cis*-isomer and the ring-closed states are quite unstable.^1^ Triphenylethylene derivatives can overcome these problems and might, therefore, be ideal fast-response photochromic systems. In addition, introducing halogen atoms into triphenylethylene derivatives can be expected to thereby enhance the ring-closing photochromic process.6a–e

Triphenylethylene derivatives are also well known as aggregation-induced emission (AIE) molecules. In contrast to the aggregation-caused quenching (ACQ) process, the emission intensities of AIE molecules are significantly increased by aggregation where the restriction of intramolecular rotation can efficiently avoid the usual emission quenching in the solid state. Since the pioneering work of Tang *et al.* in 2001,^14^ several AIE systems have been developed including silole,^15^ tetraphenylethene,^16^ and triphenylethylene derivatives^17^ and others.^18^ AIE has applications in OLED materials, piezochromic materials, chemosensors, photosensitizers and biolabels.^14^–^18^ We are aware of only one report of molecules which show both photochromic and AIE properties. During the course of our present studies, Tang *et al.* described fast responsive, photo-reversible and thermo-irreversible photochromic properties of three tetraarylethene-based polycyclic hydrocarbons which are AIE active.^19^

Triphenylethylene derivatives were first reported to be AIE active by Chi *et al.*17*a* and the piezochromic properties of these molecules have been widely investigated.17*d* We now report the first example of triphenylethylene derivatives which possess combined AIE, photochromic and photoresponsive wettability properties, namely TrPECl_2_ (Scheme S1†). As in Tang's recent report,^19^ photochromism arises due to the photocyclization of stilbene moieties in the chromophores. Compared with previous AIE photochromic system, the response times of the triphenylethylene derivatives are shortened from minutes to seconds. Therefore, these materials have advantages in the areas of optical shutters and photo-switchable molecular devices.^20^ To the best of our knowledge, there has been no previous report of an AIE-photochromic-photoresponsive wettability system with such simple structures and short response times. The straightforward synthetic route to TrPE and TrPECl_2_ is shown in Scheme S1 in the ESI.†

## Results and discussion

**Fig. 1:**
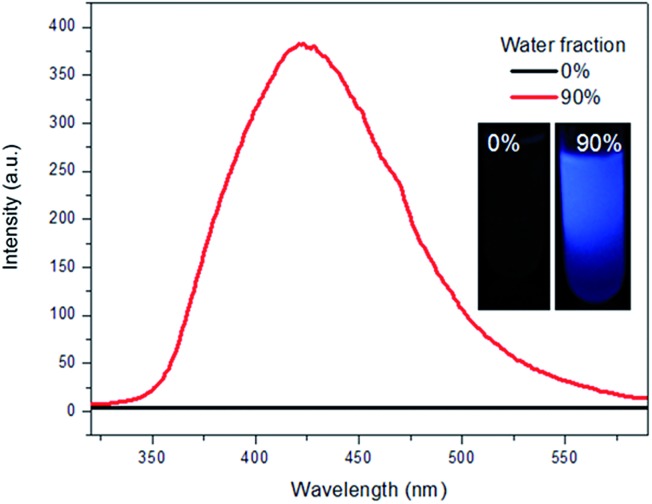
Emission spectra of TrPECl_2_ in THF/water mixed solutions containing 0% and 90% water fractions; the inset shows the photographs of TrPECl_2_ in THF/water mixed solutions containing 0% and 90% water fractions.

Triphenylethylene (TrPE) is a typical AIE unit for the construction of AIE-active molecules^17^ and compound TrPECl_2_ also shows AIE properties. AIE behavior in solution is commonly proven by the enhancement of emission, resulting from fluorescent nanoparticle formation by increasing the ratio of a poor solvent.^14^–^18^ The data for TrPECl_2_ in THF/water mixed solvent system at room temperature is shown in Fig. 1. In the pure THF solution (good solvent), the photoluminescence (PL) of the compound is not detectable. This can be attributed to the fact that intramolecular rotation, which serves as the relaxation channel for the excited state to decay, is active. However, when the water fraction reached 90% (v/v) (water is a poor solvent for TrPECl_2_), the PL intensity was boosted by many fold with an emission peak at *λ*_max_ 425 nm. This dramatic enhancement in luminescence indicates that the nanoparticles are formed in the proportion of 90 : 10 (v/v) water/THF, and that intramolecular rotation is restricted due to aggregation of the molecules, as is typically observed in AIE-active molecules. The emission band mainly originates from the π–π* transition of the triphenylethylene moieties according to previous reports.17*a* UV-vis spectra of TrPECl_2_ in THF/water mixed solutions containing 0% and 90% water fractions are shown in Fig. S2.† It is obvious that the baseline is enhanced when the water fraction reached 90%, which indicates the formation of nanoparticles.^21^

**Fig. 2:**
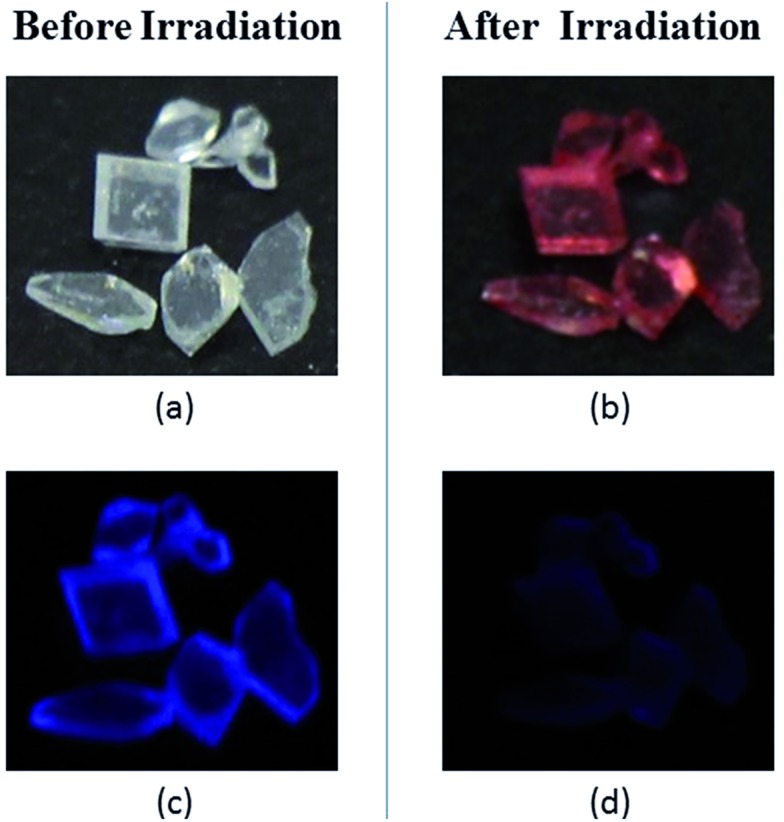
Photographs of the crystals of TrPECl_2_: (a) Before irradiation, the picture was taken under room lighting, (b) after irradiation, the picture was taken under room lighting, (c) before irradiation, the picture was taken under UV light, and (d) after irradiation, the picture was taken under UV light.

**Fig. 3:**
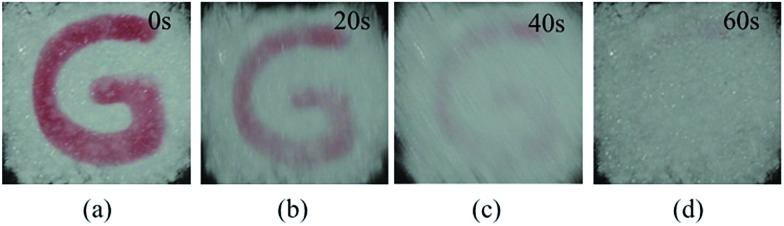
Photochromic bleaching process of TrPECl_2_ at room temperature after irradiation with UV light stopped for: (a) 0 s, (b) 20 s, (c) 40 s, and (d) 60 s.

For compound TrPECl_2_, blue emission is observed in the solid state upon excitation with UV light (365 nm), which is similar to that in AIE studies. Meanwhile, the color of the solid rapidly changes from white into bright red under UV irradiation as shown in Fig. 2(b) and the blue emission is quenched subsequently as shown in Fig. 2(d). The red solid reverts into a white solid after several seconds, and the photochromic bleaching process is described in Fig. 3. After turning to white, the emission can be observed under UV irradiation again. This cycle can be repeated many times without significant fatigue. The repeatability of the photochromic process will be discussed later. TrPE shows similar photochromic properties with shorter irradiation saturation times and photochromic bleaching times under the same conditions.

**Fig. 4:**
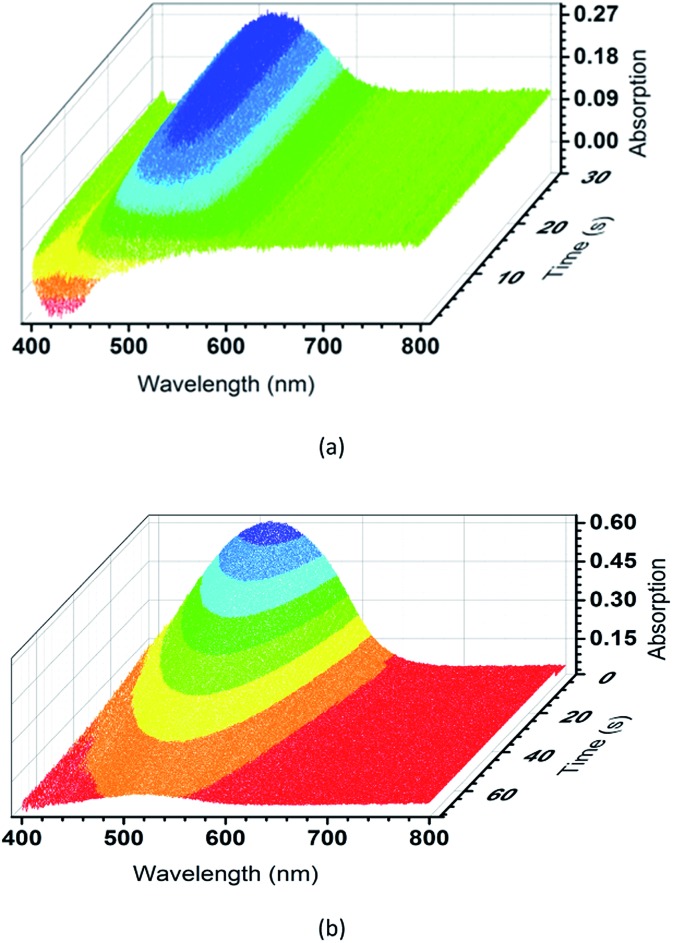
(a) Time dependent UV-vis reflectance spectra of TrPECl_2_ during the irradiation process. (b) Time dependent UV-vis reflectance spectra of TrPECl_2_ during the photochromic bleaching process.

To further evaluate the photochromic properties of TrPECl_2_ and TrPE, time dependent UV-vis reflectance spectroscopy studies were carried out: the spectra of TrPECl_2_ under irradiation are shown in Fig. 4(a). In the UV-vis reflectance spectra, the absorption bands below 400 nm are assigned as π–π* transitions, in accordance with previous studies of triphenylethylene derivatives.^17^,^22^ Before irradiation, bleaching signals with *λ*_max_ of 429 nm were also detected in the UV-vis reflectance spectra, mainly due to the emission of TrPECl_2_. As shown in Fig. 4(a), the low-energy absorption bands at around 514 nm increase with the irradiation time, suggesting increased conjugation in the molecule. Meanwhile, the bleaching signals at 429 nm gradually decrease to zero with the quenching of emission, which is in accordance with the phenomena described in Fig. 2. After 30 s, the absorption bands at 514 nm reach their maximum intensity and no increase is observed with further irradiation under a deuterium light source (irradiation saturation time). Fig. 4(b) shows the time dependent UV-vis reflectance spectra for the photochromic bleaching process of over-irradiated TrPECl_2_. The absorption bands at 514 nm decreased as soon as the irradiation stopped and disappeared after 60 s (photochromic bleaching time). It could be detected that the absorption spectra in Fig. 4(b) are broader than in Fig. 4(a). This is mainly due to the bleaching signals (emission bands of TrPECl_2_) under UV irradiation during the photochromic process. The compound TrPE shows shorter irradiation saturation time (< 1 s) and photochromic bleaching time within 1 s at ambient room temperature and lighting. The much shorter irradiation and photochromic bleaching processes are mainly due to the instability of the ring-closed structures of TrPE, which will be discussed later. It should be noted that the photochromic processes of TrPE and TrPECl_2_ are generally much faster than the previously reported AIE-photochromic systems (*ca.* 60 s).^19^ The increase and decrease of the reflectance of TrPECl_2_ and TrPE at 515 nm as a function of time are shown in Fig. 4(a and b) and Fig. S5,† respectively. The repeated photochromic switching processes of TrPECl_2_ are shown in Fig. S6† for 20 cycles without fatigue. Good repeatability of these photochromic materials indicates their potential applications as rewritable materials. It is obvious from these data that triphenylethylene derivatives show both fast-response properties and high stabilities in the photochromic processes.

**Fig. 5:**
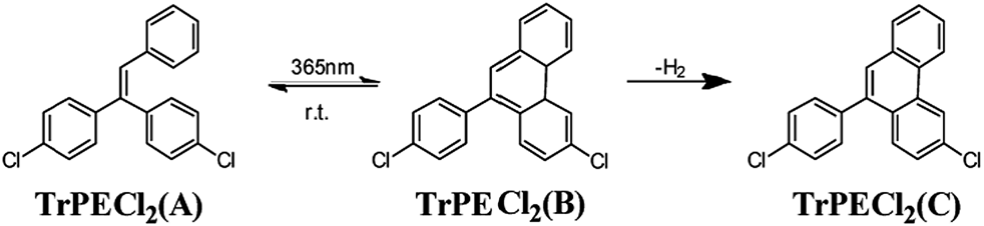
Proposed mechanism of the photochromism of TrPECl_2_.

In the triphenylethylene derivatives, the distance between the phenyl rings and the ethene-1,1-diyldibenzene moieties are short enough for ring-closure reactions.^17^,^22^ The photochromic response after irradiation is assigned to a stilbene-type 6-π electron ring-closure as depicted in Fig. 5. The ring-closure of similar structures has been reported in previous studies.^17^,^22^ In contrast to the previous report on AIE-photochromic materials,^19^ the structure of TrPECl_2_(B) is unstable and quickly reverted to structure TrPECl_2_(A) even in the dark at room temperature. The instability of the TrPECl_2_(B) structure indicates a lower energy barrier during the photochromic process, which is in accordance with the less extended π structure compared with the previously reported system.^23^ After irradiation for many cycles in air, the white solid TrPECl_2_(A) became a pale yellow color. This solid is weakly emissive in solution which is different from pure compounds TrPECl_2_(A) and TrPECl_2_(B), which are non-emissive in solution. According to a previous study, the yellow colour after irradiation is mainly due to the dehydrogenation process of compound TrPECl_2_(B) in the presence of oxygen to afford TrPECl_2_(C) (Fig. 5).^19^,^23^ However, the photochromic processes of these compounds were not observed in solution states. This is mainly attributed to the twisted structures of these AIE molecules in solution states that resist the ring-closure reaction. Similar results were also reported in AIE photochromic materials in previous studies.^19^

In an attempt to provide evidence for structure TrPECl_2_(C), a photo-oxidation reaction was performed: compound TrPECl_2_ was irradiated for 48 h in the solid state in the presence of air. The pale yellow solid was formed, which was proposed to be the dehydrogenated product TrPECl_2_(C). The ^1^H NMR spectrum of this solid showed additional peaks in the aromatic region compared to compound TrPECl_2_(A), consistent with the formation of the dehydrogenated phenanthrene derivative TrPECl_2_(C) as shown in Fig. S7 and S8 in the ESI.† To further confirm the structure of TrPECl_2_(C), a small amount of TrPECl_2_(C) was purified from the irradiated product using silica gel column chromatography with hexane as eluent. The high resolution EI mass spectrum of TrPECl_2_(C) is shown in Fig. S9† and offers validation.

**Fig. 6:**
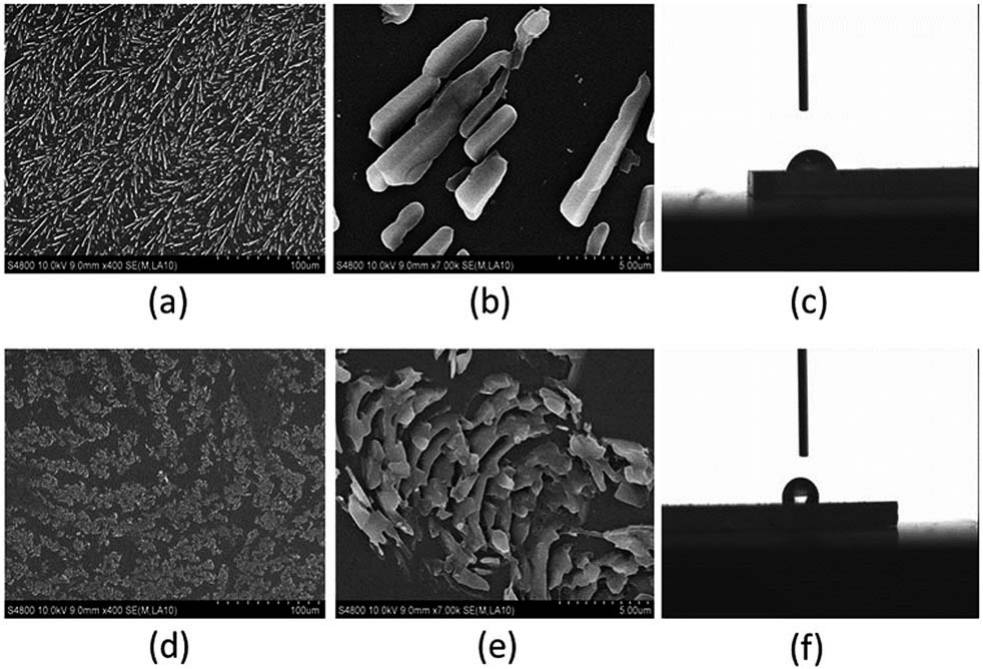
Surface morphology control of TrPECl_2_ by irradiation: (a and b) SEM image of surface A (without irradiation); (c) contact angle of the water droplet on surface A; (d and e) SEM image of surface B (with irradiation); (f) contact angle of the water droplet on surface B.

The TrPECl_2_ microcrystalline surfaces were initially prepared by spin-coating a TrPECl_2_ dichloromethane solution (5 mg mL^–1^) onto two identical pieces of SiO_2_ substrate. After the solvent was evaporated under vacuum at room temperature, one TrPECl_2_ microcrystalline surface (surface A) was formed after storage in the dark for 3 days at ambient temperature (around 303 K). The other microcrystalline surface (surface B) was formed by irradiation under UV light (365 nm) for 3 minutes and followed by storage in the dark for 3 days at room temperature. Scanning electron microscopy (SEM) and contact angle analysis studies were carried out to establish the morphology of TrPECl_2_ microcrystalline surfaces without or with irradiation. The SEM images without or with irradiation are displayed in Fig. 6(a and b) and (d and e), respectively. On surface A, without the 3 minutes irradiation, microcrystals of TrPECl_2_ with rod-shape-crystals gradually grew in the dark. A branched pattern was formed on the surface by the rod-shape-crystals as shown in Fig. 6(a). The contact angle of a water droplet on surface A is 73° (Fig. 6(c)). However, the microcrystals of TrPECl_2_ on surface B grew into a different pattern of scale-shaped crystals (Fig. 6(d and e)) and the contact angle of a water droplet on surface B increases from 73° to 118° (Fig. 6(f)). These results demonstrate that irradiation strongly affects the patterns of TrPECl_2_ microcrystals and further controls the wettability of surfaces. The topographical change of TrPECl_2_ microcrystals on the surface is mainly attributed to the formation of closed-ring isomer TrPECl_2_(B) as nuclei at the beginning of crystal growth; a similar mechanism of the topographical change has been reported previously.10d,^12^ The TrPECl_2_(B) could be changed back to TrPECl_2_(A) quickly in the crystal-growth process in the dark as mentioned above. It should be noted that these two surfaces with different patterns are formed with the same isomer [TrPECl_2_(A)] of the photochromic material that is very different from the topographical change and wettability-controllable systems based on diarylethene derivatives.^8^–^12^ These new topographically-changeable and wettability-controllable surfaces are much more stable in the light because the different microcrystalline patterns are all formed by the stable isomer of the photochromic process. In addition, the photoresponsive patterning process requires much shorter irradiation time compared with the same process for diarylethene derivatives (usually several hours).^8^–^12^ These advantages could promote these topographically-changeable and wettability-controllable materials based on triphenylethylene for industrial applications.

## Conclusions

In summary, a structurally-simple triphenylethylene derivative TrPECl_2_ has been successfully designed and synthesized and it provides a rare example of combined AIE, fast-responsive photochromism, and topographically-changeable and wettability-controllable properties in a single compound. To study the photochromic properties of these materials in the solid state, UV-vis reflectance spectroscopy studies have been performed. SEM and contact angle analysis have been used to investigate the morphology and wettability control of the TrPECl_2_ microcrystalline surfaces by irradiation. These new compounds enlarge our understanding of the photophysical and photochemical properties of triphenylethylene derivatives and present a new strategy to design multifunctional materials with simple structures.

## Supplementary Material

Supplementary information

## References

[cit1] Kellogg R. M., Greon M. B., Wynberg H. (1967). J. Org. Chem..

[cit2] Tsivgoulis G. M., Lehn J.-M. (1995). Angew. Chem., Int. Ed..

[cit3] (b) Dürr H. and Bouas-Laurent T. H., Photochromism: Molecules and Systems, Elsevier, Amsterdam, 1990.

[cit4] (a) Hovey R. J., Fuchsman C. H., Chu N. Y. C. and Piusz P. G., photochromic compound, US Pat., 4215010, 1980.

[cit5] Tamai N., Miyasaka H. (2000). Chem. Rev..

[cit6] Irie M., Mohri M. (1988). J. Org. Chem..

[cit7] Broman S. L., Nielsen M. B. (2014). Phys. Chem. Chem. Phys..

[cit8] Nakamura S., Yokojima S., Uchida K., Tsujioka T. (2011). J. Photochem. Photobiol., C.

[cit9] Kitagawa D., Kobatake S. (2012). Chem. Sci..

[cit10] Uchida K., Nishikawa N., Izumi N., Yamazoe S., Mayama H., Kojima Y., Yokojima S., Nakamura S., Tsujii K., Irie M. (2010). Angew. Chem., Int. Ed..

[cit11] Kitagawa D., Yamashita I., Kobatake S. (2010). Chem. Commun..

[cit12] Oropesa-Nuñez R., Fragouli D., Athanassiou A. (2014). Langmuir.

[cit13] Fragouli D., Athanassiou A. (2008). Adv. Funct. Mater..

[cit14] Luo J., Xie Z., Lam J. W. Y., Cheng L., Qiu C., Kwok H. S., Zhan X., Liu D., Zhu D., Tang B. Z. (2001). Chem. Commun..

[cit15] Yu G., Yin S., Liu Y., Chen J., Xu X., Sun X., Ma D., Zhan X., Peng Q., Shuai Z., Tang B. Z., Zhu D., Fang W., Luo Y. (2005). J. Am. Chem. Soc..

[cit16] Dong Y., Lam J. W. Y., Qin A., Liu J., Li Z., Tang B. Z., Sun J., Kwok H. S. (2007). Appl. Phys. Lett..

[cit17] Yang Z., Chi Z., Yu T., Zhang X., Chen M., Xu B., Liu S., Zhang Y., Xu J. (2009). J. Mater. Chem..

[cit18] Zheng Y. S., Hu Y. J. (2009). J. Org. Chem..

[cit19] He Z., Shan L., Mei J., Wang H., Lam J. W. Y., Sung H. H. Y., Williams I. D., Gu X., Miao Q., Tang B. Z. (2015). Chem. Sci..

[cit20] Wirnsberger G., Scott B. J., Chmelka B. F., Stucky G. D. (2000). Adv. Mater..

[cit21] Tang B. Z., Geng Y., Lam J. W. Y., Li B., Jing X., Wang X., Wang F., Pakhomov A. B., Zhang X. (1999). Chem. Mater..

[cit22] Zhou X., Li H., Chi Z., Xu B., Zhang X., Zhang Y., Liu S., Xu J. (2012). J. Fluoresc..

[cit23] Navale T. S., Thakur K., Rathore R. (2011). Org. Lett..

