# Supplementary material for: Combined aggregation induced emission (AIE), photochromism and photoresponsive wettability in simple dichloro-substituted triphenylethylene derivatives
Source: Chem Sci. 2016 Apr 26;7(8):5302–6. doi: 10.1039/c6sc01205a (PMC6020526; doi:10.1039/c6sc01205a)
Supplement: Supplementary file 1 [file SC-007-C6SC01205A-s001.pdf]

Electronic Supplementary Material (ESI) for Chemical Science.
This journal is © The Royal Society of Chemistry 2016

Supporting Information

1 Synthesis of TrPE and $TrPECl_2$

The compounds TrPE and $TrPECl_2$ were synthesized according to the synthetic routes described in Scheme S1. The details of the synthetic procedure for TrPE was according to previous literature[1] and the details of synthetic procedure of $TrPECl_2$ was listed below. These compounds were characterized by ^{1}H NMR spectroscopy, EI mass spectroscopy.

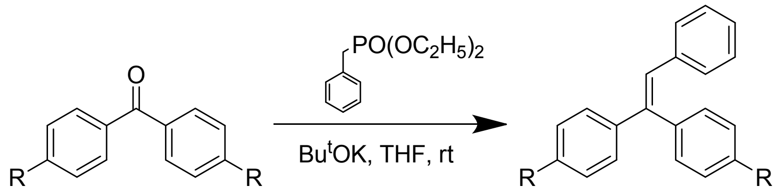

TrPE: R = H **$TrPECl_2$:** R = Cl

Scheme S1 Synthetic routes for TrPE and $TrPECl_2$

$TrPECl_2$. To a two-necked round-bottomed flask containing bis(4-chlorophenyl) methanone (0.502 g, 2.0 mmol) and diethyl benzylphosphonate (0.456 g, 2.0 mmol) was added degassed THF (30 mL) under an argon atmosphere. After cooling to 0 ^{o}C, ^{t}BuOK (0.224 g, 2 mmol) was added. Upon stirring for 12 hours under an argon atmosphere, the mixture was poured into 95% ethanol (150 mL), and stirred for another 1 hour. The white precipitate which formed was collected by filtration. The precipitate was dissolved in CH_2Cl_2 and washed 3 times with water. The organic layer was dried over anhydrous $MgSO_4$. After evaporation of the filtrate, the residue was purified by recrystallization via vapor diffusion of hexane into a concentrated dichloromethane solution of the product to give $TrPECl_2$ as a white powder. Yield: 0.500 g (77 %). ^{1}H NMR (400 MHz, $CDCl_3$, 298 K): δ = 6.95 (s, 1H; –CH=), 7.03–7.05 (m, 2H; $–C_6H_5$), 7.11–7.24 (m, 7H; $–C_6H_5$, $–C_6H_4–$), 7.28–7.32 (m, 4H; $–C_6H_4–$); High Resolution EI–MS: m/z found: 324.0470 $[M]^+$; calcd for $C_{20}H_{14}Cl_2$: 324.0473. Anal. calcd for $C_{20}H_{14}Cl_2$: C 73.86, H 4.34, Cl 21.80; found: C 73.81, H 4.37.

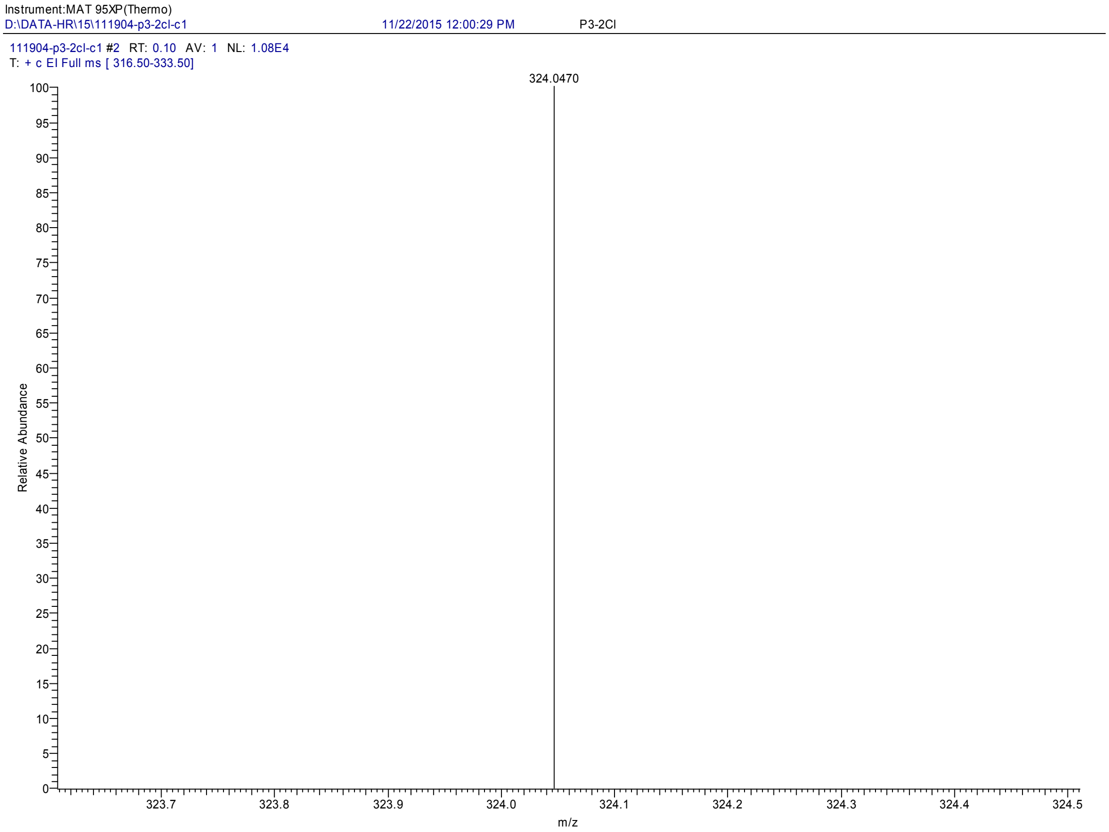

Figure S1 High resolution EI mass spectrum of $TrPECl_2$.

2 Physical measurements and instrumentations

^1H NMR spectra were obtained on a Varian Mercury-Plus 300 Nuclear Magnetic Resonance Spectrometer with chemical shifts recorded relative to tetramethylsilane (Me_4Si). Positive ion EI mass spectra were recorded on a Thermo MAT95XP high resolution mass spectrometer. UV-vis reflectance spectra were recorded on an Ocean Optic Maya2000PRO spectrometer with Ocean Optic reflection probes R600-125F. UV light source for the irradiation process was Ocean Optic D-2000 deuterium lamp with 536 μW output powers, and the emission wavelengths were ranging from 215 nm to 400 nm. UV light source for the photochromic bleaching process and photoresponsive wettability studies was Ocean Optic LLS365 LED lamp with the emitting wavelength of 365 nm. Half band width of the light source was 9 nm and output power of the light source was 2.5 mW. Steady state emission spectra were recorded using a Shimadzu RF-5301pc spectrofluorophotometer.

3 Photochromism Figures

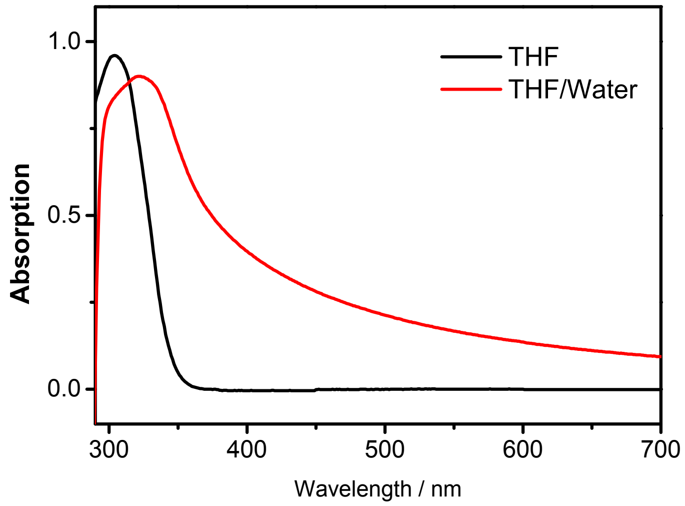

Figure S2 UV-vis spectra of $TrPECl_2$ in the THF/water mixed solutions containing 0 % and 90 % water fraction.

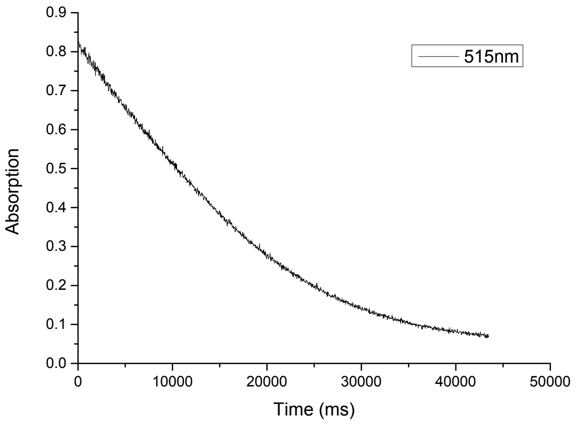

Figure S3 Time dependent reflectance decay curve at 515 nm of $TrPECl_2$ during the photochromic bleaching process.

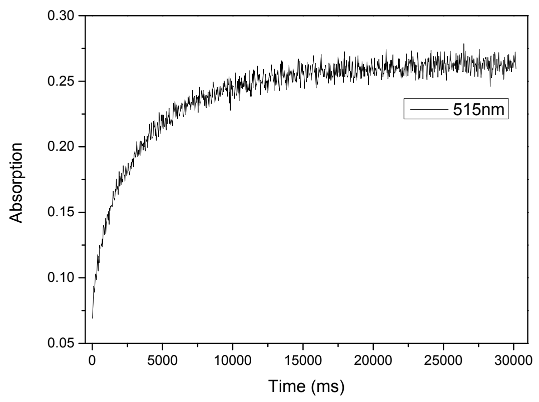

Figure S4 Time dependent reflectance saturating curve at 515 nm of $TrPECl_2$ during the photochromic saturation process.

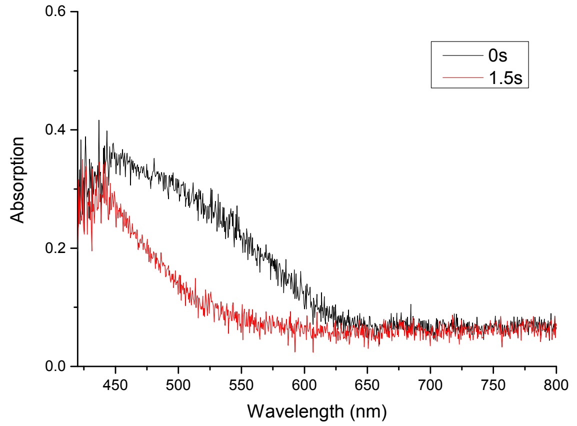

Figure S5 UV-vis reflectance spectra of TrPE with 0 s and 1.5 s after LED UV light off.

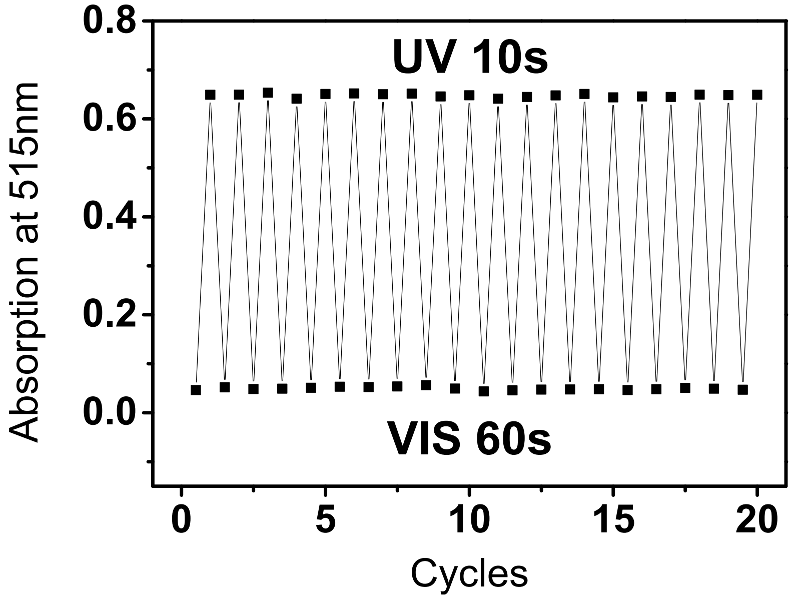

Figure S6 Recycling of the photochromic process of compound $TrPECl_2$ as a function of exposure to UV (365 nm) and ambient room light for 10 and 60 s, respectively.

4 ^{1}H NMR and mass spectra of $TrPECl_2$(C)

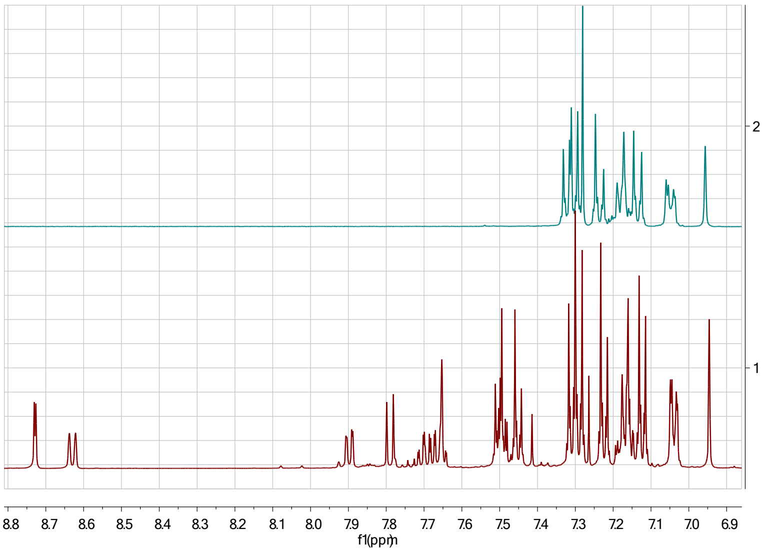

Figure S7 ^{1}H NMR spectra of $TrPECl_2$**(A)** and proposed product $TrPECl_2$**(A)** mixed with $TrPECl_2$**(C).**

^{1}H NMR spectrum of TrPECl$_2$**(C)** was assigned as shown in Figure S8. To further prove the mechanism, trace amount of TrPECl$_2$**(C)** was received by column chromatography with hexane as eluent. High resolution EI mass spectrum of TrPECl$_2$**(C)** was carried as shown in Figure S9. ^{1}H NMR spectrum of TrPECl$_2$**(C)** was listed as following. Assignment was made according to similar structures reported in previous literatures.[2] .^{1}H NMR (400 MHz, $CDCl_3$, 298 K): δ = 7.44-7.53 (m, 5H; $-C_6H_4-$ and phenanthrene), 7.66–7.72 (m, 3H; phenanthrene), 7.81 (d, 8.84 Hz, 1H; phenanthrene), 7.92 (d, 7.60 Hz, 1H; phenanthrene), 8.65 (d, 8.00 Hz, 1H; phenanthrene), 8.75 (s, 1H; phenanthrene) High resolution EI: m/z 322.0312 $[M]^+$; calcd for $C_{20}H_{12}Cl_2$: 322.0316.

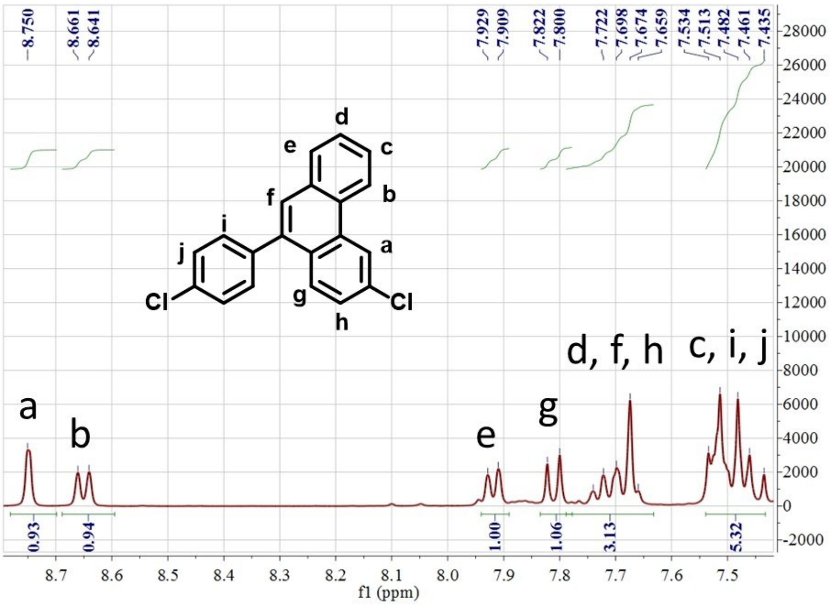

Figure S8 ^{1}H NMR spectrum and assignment of TrPECl$_2$**(C)**.

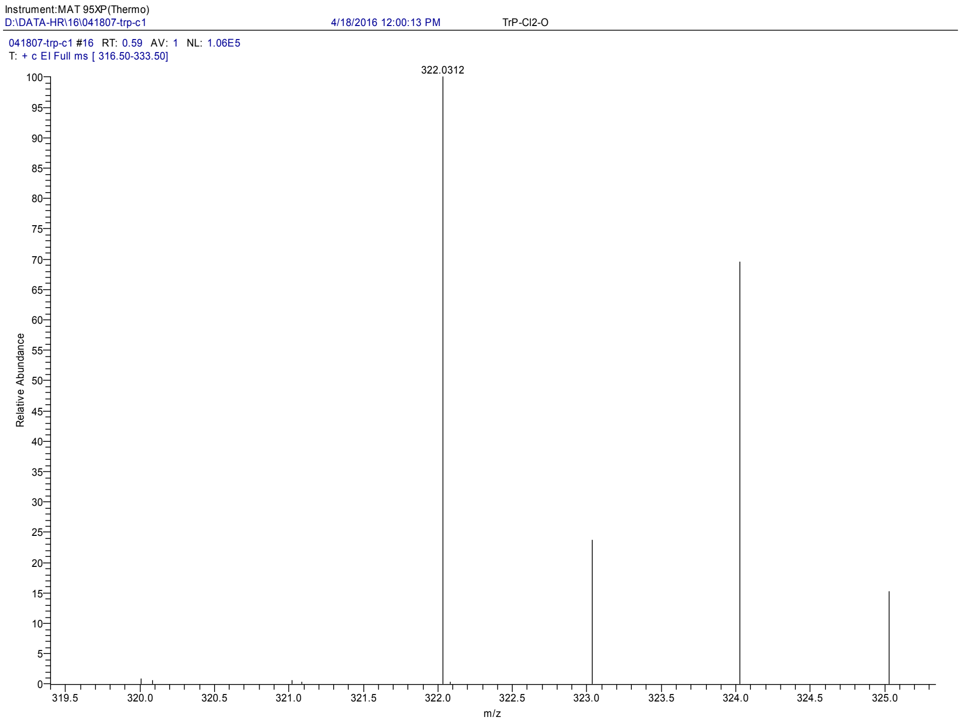

Figure S9 High resolution EI mass spectrum of $TrPECl_2$**(C).**

References

[1] R. Baker and R. J. Sims, *Synthesis*, 1981, **2**, 117.

[2] a) D. García-Cuadrado, P. de Mendoza, A. A. C. Braga, F. Maseras and A. M. Echavarren, *J. Am. Chem. Soc.*, 2007, **129**, 6880; b) K. Funaki, H. Kawai, T. Sato, and S. Oi, *Chem. Lett.,* 2011, **40**, 1050.
